# MAD2B-mediated cell cycle reentry of podocytes is involved in the pathogenesis of FSGS

**DOI:** 10.7150/ijbs.62238

**Published:** 2021-10-22

**Authors:** Dian Bao, Hua Su, Chun-Tao Lei, Hui Tang, Chen Ye, Wei Xiong, Fang-Fang He, Ji-Hong Lin, Hans-Peter Hammes, Chun Zhang

**Affiliations:** 1 Department of Nephrology, Union Hospital, Tongji Medical College, Huazhong University of Science and Technology, Wuhan 430022, China.; 2 5th Medical Department, Medical Faculty Mannheim, University of Heidelberg, D-68167 Mannheim, Germany.

**Keywords:** podocyte, FSGS, cell cycle reentry, MAD2B, ATM

## Abstract

**Rationale:** Focal segmental glomerulosclerosis (FSGS) is characterized by the dysfunction of “post-mitotic” podocytes. The reentry of podocytes in the cell cycle will ultimately result in cell death. Mitotic arrest deficient 2-like protein 2 (MAD2B), an inhibitor of anaphase-promoting complex (APC)/cyclosome, precisely controls the metaphase to anaphase transition and ordered cell cycle progression. However, the role of MAD2B in FSGS podocyte injury remains unknown.

**Methods:** To explore MAD2B function in podocyte cell cycle reentry, we used conditional mutant mice lacking MAD2B selectively in podocytes in ADR-induced FSGS murine model. Additionally, KU-55933, a specific inhibitor of ataxia-telangiectasia mutated (ATM) was utilized *in vivo* and *in vitro* to explore the role of ATM in regulating MAD2B.

**Results:** The expression of MAD2B in podocytes was dramatically increased in patients with FSGS and ADR-treated mice along with podocyte cell cycle reentry. Podocyte-specific knockout of MAD2B effectively attenuated proteinuria, podocyte injury, and prevented the aberrant cell cycle reentry. By bioinformatics analysis we revealed that ATM kinase is a key upstream regulator of MAD2B. Furthermore, inhibition of ATM kinase abolished MAD2B-driven cell cycle reentry and alleviated podocyte impairment in FSGS murine model. *In vitro* studies by site-directed mutagenesis and immunoprecipitation we revealed ATM phosphorylated MAD2B and consequently hampered the ubiquitination of MAD2B in a phosphorylation-dependent manner.

**Conclusions:** ATM kinase-MAD2B axis importantly contributes to the cell cycle reentry of podocytes, which is a novel pathogenic mechanism of FSGS, and may shed light on the development of its therapeutic approaches.

## Introduction

Focal segmental glomerulosclerosis (FSGS) is a common pathologic type of refractory nephrotic syndrome and its underlying etiologies are heterogeneous, such as circulating factors, gene mutation, infection, and toxicity or adaptive responses. Up to now, effective therapeutic measures are still limited and more than 50% of patients with FSGS who respond poorly to conventional therapies will progress to end-stage renal disease 1-3. It is well-accepted that morphological alterations of podocyte with the eventually cell death is the core event during FSGS no matter its original causes 4-6. Thus, to explore the mechanisms for podocyte injury in FSGS lesions is crucial.

The strict cell cycle control is vital for terminally differentiated cells, including podocytes. Physiologically, podocytes exit from the cell cycle and remain at the G0 phase. However, cell cycle reentry of podocytes is observed in several kidney diseases including FSGS, diabetic kidney disease and viral infection 7, 8. Since podocytes possess a unique cytoskeletal architecture, they cannot complete replication and maintain their actin cytoskeleton during mitosis 9, which usually results in podocyte death and detachment from the kidney glomerular basement membrane (GBM) instead of normal division. Therefore, the mitotic catastrophe, a unique type of cell death caused by improper mitosis, of podocytes with underlying aberrant cell cycle is the core pathologic event during FSGS.

The anaphase-promoting complex/cyclosome (APC/C) acts as a large multiprotein E3 ligase and regulates the orderly mitotic events in cells, which is controlled by APC/C activating subunits such as Cadherin (Cdh)1 and cell division cycle 20 (CDC20). Mitotic arrest deficient 2-like protein 2 (MAD2L2, also known as MAD2B or REV7) belongs to the MAD gene family that controls DNA repair, mitotic spindle checkpoint 10, cell cycle regulation 11, 12 and tumorigenesis 13. MAD2B suppresses the activity of Cdh1 and CDC20 in the G1 phase of mitosis, resulting in APC/C incapacity 12. As an E3 ubiquitin ligase, APC/C could mediate the ubiquitination and degradation of several vital cell cycle regulators, including cyclin B1 and S-phase kinase-associated protein 2 (Skp2), and play a key role in mitotic events. Previously, we reported that MAD2B is upregulated in diabetic nephropathy and largely contributes to podocyte injury by inhibiting APC/C-mediated cyclin B1 and Skp2 degradation 14. In the present study, using podocyte-specific MAD2B knockout mice and *in vitro* cultured cells, we explored the role of MAD2B in cell cycle regulation of podocytes and expected to get further understanding of the pathogenesis of FSGS.

## Materials and methods

### Study approval

The sampling procedures were conducted in accordance with the Declaration of Helsinki and were approved by the Ethics Committee of Huazhong University of Science and Technology, and informed consent was obtained from all the patients. All animal experimental procedures in this study were approved by the Ethics Committee of Huazhong University of Science and Technology and in compliance with guidelines for animal care.

### Human renal biopsy samples

According to the Columbia classification, not otherwise specified (NOS) FSGS cases without detected secondary etiologies were enrolled in this study. The FSGS tissues were sampled by the Department of Nephrology, Union Hospital, Tongji Medical College, Huazhong University of Science and Technology. The control samples were obtained from the para-carcinoma renal tissues of patients who underwent tumor nephrectomy without any primary or secondary renal diseases.

### ADR-induced FSGS murine model

Adult male mice (8 wks of age with Balb/C background) weighing 21-24 g were obtained from Charles River Laboratories (Beijing, China), and raised in a specific pathogen-free environment with a 12 h light/dark cycle, and allowed access to food and water* ad libitum*. To establish the FSGS animal model, the mice were injected with a single dose of ADR (15 mg/kg) via the tail vein and sacrificed after 4 wks. Urine and serum samples were harvested prior to sacrifice, and the urine protein/creatinine, serum albumin, creatinine, and blood urea nitrogen were measured using an automated chemistry analyzer. After flushing with ice-cold Krebs-Henseleit-saline buffer via an aortal catheter, the kidneys were dissected on ice. The cortex tissues were snap frozen and stored at -80°C until use. To inhibit ATM kinase, KU-55933 (500 µg/kg; Selleck Chemicals, Houston, TX, USA), a specific ATM inhibitor, was dissolved in 0.15% DMSO and was administered intraperitoneally 24 h prior to ADR injection and repeated every 3 days until sacrifice.

### Generation of podocyte-specific MAD2B knockout mice

MAD2B*^fl/fl^* mice with C57BL/6 background were obtained from the Shanghai Model Organisms Center (Shanghai, China). Then, MAD2B*^fl/fl^* mice were crossed with C57BL/6 mice expressing Cre recombinase (Cre) under the control of the podocin promoter (*Nphs2.Cre*; JAX 008205, Jackson Laboratory, ME, USA). Then, the MAD2B*^+/fl^* mice were crossed with *Nphs2.Cre^+^* mice, and double heterozygous mice were backcrossed to generate podocyte-specific knockout mice (Podocin-Cre MAD2B*^fl/fl^* mice; Cre^+^/MAD2B*^fl/fl^* mice). MAD2B*^fl/fl^* littermates without Cre expression were used as WT controls (Cre^-^/MAD2B*^fl/fl^* mice). Samples were obtained from the mice tails at 2 wks of age for genotyping and PCR. To establish the FSGS model, adult mice (12 wks of age) from both sexes were injected with a single dose of ADR (18 mg/kg) via the tail vein and then sacrificed 6 wks later.

### Histopathological assessment

Periodic acid-Schiff (PAS) staining of 4 μm paraffin sections was used to assess the morphological changes in the glomeruli of different groups. Fifty randomly selected glomeruli from each section were analyzed to obtain the glomerular damage index (GDI) score as described previously 15, 16.

### Transmission electron microscopy

Electron microscopic sample handling and detection were performed by the core lab of the Wuhan Institute of Virology. The images were obtained using a transmission electron microscope (Tecnai G^2^ 20 TWIN, FEI Company, PA, USA).

### Immunostaining of kidney sections

After fixation and permeabilization, the tissue sections were blocked with 5% donkey serum and then incubated overnight at 4°C with the following primary antibodies: anti-synaptopodin antibody (1:300; 163004, Synaptic system), rabbit monoclonal anti-Skp2 (1:50; ab183039, Abcam, Cambridge, MA, USA), rabbit polyclonal anti-podocin (1:50; p0372, Sigma-Aldrich, St. Louis, MO, USA), and rabbit anti-MAD2B (1:100; ab180579, Abcam, Cambridge, MA, USA). After rinsing with PBS thrice, the slides were incubated with Alexa 488- or Alexa 647-labeled secondary antibodies at room temperature for 1 h. Images were captured using a laser-scanning confocal microscope (LSM 710, Carl Zeiss, Jena, Germany) at identical microscope settings.

For IHC staining of MAD2B, Ki67, Wilms tumor gene (WT1) and pATM in biopsy samples, the slides were incubated at 60°C for 30 min followed by deparaffinization and rehydration. Sections of mice kidney tissues were incubated with anti-MAD2B (1:100; ab180579, Abcam, Cambridge, MA, USA), WT1 (1:200, ab89901, Abcam, Cambrige, MA, USA) or anti-pATM antibody (1:50; 13050, Cell Signaling Technology, Danvers, MA, USA) overnight at 4°C. For human biopsy slides, anti-MAD2B (1:300, Rockland, Gilbertsville, PA) and anti-Ki67 (1:200, ab16667, Abcam, Cambridge, MA, USA) were utilized. The sections were then washed in PBS and incubated with biotinylated secondary antibody and avidin-biotin peroxidase complex (Dako, Shanghai, China) for 20 min. Finally, the peroxidase activity was visualized using diaminobenzidine, and sections were counterstained with hematoxylin and observed under a light microscope. Twenty randomly selected glomeruli were analyzed using Image-Pro Plus 5.10 software (Media Cybernetics, Rockville, MD, USA). Integrated optical density was used to indicate the relative levels of MAD2B and pATM expressions. In fifty glomeruli/kidney, the number of Ki67^+^ and WT1^+^ cells of the entire glomerulus were counted (magnification×400).

### Cell culture, treatment, and transfection

Conditional immortalized human podocytes were cultured and allowed to differentiate as previously described 17, and then they were manipulated with following experimental procedures. Treatment with ADR (0.4 μg/ml; Aladdin, Shanghai, China) for different time periods was utilized to setup podocyte insult. Pretreatment with KU-55933 (10 μM; Selleck, Shanghai, China) was applied to inhibit ATM kinase activation. Knockdown of MAD2B was achieved by lentiviral transfection according to the manufacturer's instructions. MAD2B shRNA was from GeneChem (Shanghai, China), and scrambled shRNA was applied as control. Lentiviral vectors for His-tagged MAD2B (NCBI reference sequence ID, NM_006341) overexpression and mutagenesis were purchased from Qijing Biochemical Co., Ltd (Wuhan, China). After 10 hours of incubation, the medium was completely exchanged.

### Western blotting and immunoprecipitation analyses

Briefly, total cell lysates were prepared with radioimmunoprecipitation assay (RIPA) Lysis Buffer (Beyotime Biotechnology, Shanghai, China) containing protease inhibitors. The western blot analysis was performed as follows: after determination of protein concentration using the bicinchoninic acid (BCA) Protein Assay Kit (Beyotime Biotechnology) and denaturation, an equal amount of protein (50 µg) was loaded and separated by SDS-PAGE and transferred to PVDF membranes. The membranes were blocked for 1 h and then were incubated with the indicated primary antibodies overnight at 4°C. The primary antibodies were listed below. MAD2B (1:800, 12683-1-AP), cyclin B1 (1:1000, 55004-1-AP), Skp2 (1:1000, 15010-1-AP), p27 (1:1000, 25614-1-AP), GAPDH (1:15000, 60004-1-Ig), α-tubulin (1:10000, 11224-1-AP), His-tag (1:5000, 66005-1-Ig) and ubiquitin (1:1000, 10201-2-AP), which were all purchased from Proteintech. CD2AP (1:500, BS70918) were from Bioworld Technology (Louis Park, MN, USA), Nephrin (1:1000, ab136894) were from Abcam, ATM (1:1000, 2873), pATM (1:500, 13050), and phospho-(Ser/Thr) ATM/ATR Substrate (1:1000, 2851) were from Cell Signaling Technology. Afterwards the membranes were washed with TBS with 0.1% Tween 20 (pH 7.2-7.6) and incubated with the corresponding HRP-conjugated secondary antibodies at room temperature for 1 h. The signals from the ECL reagent reaction were visualized and developed on films. The relative intensity of the target bands was quantified using the ImageJ software. All data of protein expressions and the ratio of pATM/ATM were analyzed as the folds versus the control/vehicle group after normalization.

For immunoprecipitation, the cell lysates were prepared as described above. Equal quantities (1-1.5 mg) of proteins were separated and supplied with 1µg anti-MAD2B (12683-1-AP, Proteintech, Chicago, IL) and 30 µL Protein A/G PLUS-Agarose (Santa Cruz Biotechnology, Santa Cruz, CA, USA). For cell lysates of lentiviral vector-mediated His-tagged MAD2B overexpression and mutagenesis, anti-His magnetic beads (Beyotime Biotechnology) were utilized to prepare immunoprecipitates according to the manufacturer's instructions. The complex was incubated on a rotary device (Liuyi Biotechnology, Beijing, China) overnight at 4°C. The next day, the immunoprecipitates were collected by centrifugation at 560g for 5 min at 4°C. After three rinses with PBS, the pellets were resuspended in SDS loading buffer, boiled at 98°C for 5 min, and analyzed by western blotting.

### Cell cycle analysis

The cell cycle status was quantitatively analyzed by flow cytometry. After the indicated treatment, podocytes were collected and fixed in cold 70% ethanol overnight at 4°C. Next, the podocytes were incubated with propidium iodide (50 μg/ml) containing 0.1 mg/ml RNase A and 0.2% Triton X-100 for 30 min at 37°C. Then, the DNA content was analyzed using BD LSR Cell Analyzer (Becton Dickinson, Franklin Lakes, NJ, USA) to quantify the proportion of cells in each cell cycle phase, and 10,000 gated events were acquired per sample. Analysis of cell cycle distribution was performed with FlowJo software (FlowJo, Ashland, USA).

### Statistics

All data in this study were recorded as the mean± SEM. Significant differences between two groups were analyzed using a two-tailed *t*-test. Multiple-group comparisons were evaluated using one-way ANOVA followed by Dunnett's multiple comparison tests with GraphPad Software 7.0.* P*<0.05 was considered to indicate statistical significance. Particular tests performed in the experiments are indicated in the figure legends.

## Results

### MAD2B is elevated in glomeruli from FSGS lesions and ADR-treated podocytes

**Figure 1:**
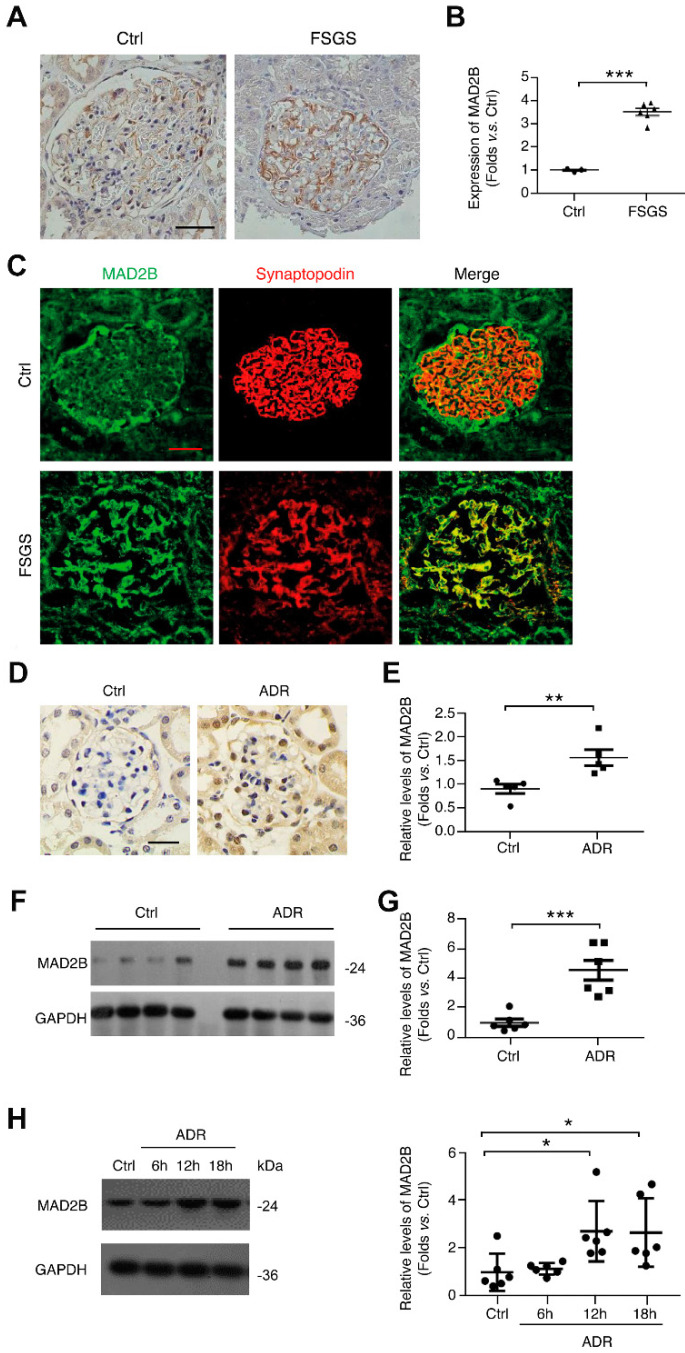
** The expression of MAD2B is elevated in glomeruli from FSGS patients and ADR mice, as well in ADR-treated podocytes. (A, B)** Representative photomicrograph of MAD2B density in the glomeruli of biopsies from patients with FSGS (Scale bar=25μm) and summarized data indicating MAD2B protein level; *n* = 6. **(C)** Representative confocal images of immunofluorescence staining of MAD2B and synaptopodin in the glomeruli of patients with FSGS (Scale bar=25μm). **(D, E)** Representative images of MAD2B IHC staining in the glomeruli of ADR-induced FSGS mouse model (Scale bar=25μm) and the summarized data indicating MAD2B protein level; *n* = 6. **(F, G)** Representative western blot images and summarized data indicating MAD2B protein level in the cortex of mice kidneys in each group; *n* =6. (H) Representative western blot images and summarized data showing MAD2B expression in podocytes treated with 0.4 μg/ml ADR for indicated times; *n* =6. Ctrl: control; ADR: Adriamycin; *P<0.05 vs. Ctrl; **P<0.01 vs. Ctrl; ***P<0.001 vs*.* Ctrl. **(B, E and G)** Statistical analysis was performed using two-tailed Student's *t*-test. **(H)** Statistical analysis was performed using one-way ANOVA with Dunnett's correction. MAD2B, mitotic arrest deficient-like 2B; FSGS, focal segmental glomerulosclerosis.

Immunohistochemical (IHC) staining was used to observe the expression of MAD2B in renal biopsy tissues from patients with FSGS. Figure 1A and B show that the intensity of MAD2B staining was significantly enhanced in glomeruli of patients with FSGS (*P*<0.001). Immunofluorescence (IF) staining and confocal microscopy confirmed that the elevated MAD2B was colocalized with synaptopodin, a podocyte marker (Figure 1C). Similarly, the level of MAD2B was also elevated in the glomeruli of experimental FSGS mice in IHC (*P*<0.01) and western blot (WB) (*P*<0.001) studies (Figure 1D-G). In addition, the level of MAD2B was evidently enhanced (*P*<0.05) in podocytes under ADR stimulation (Figure 1H).

### ADR induces cell cycle reentry in both murine FSGS model and cultured podocytes

**Figure 2:**
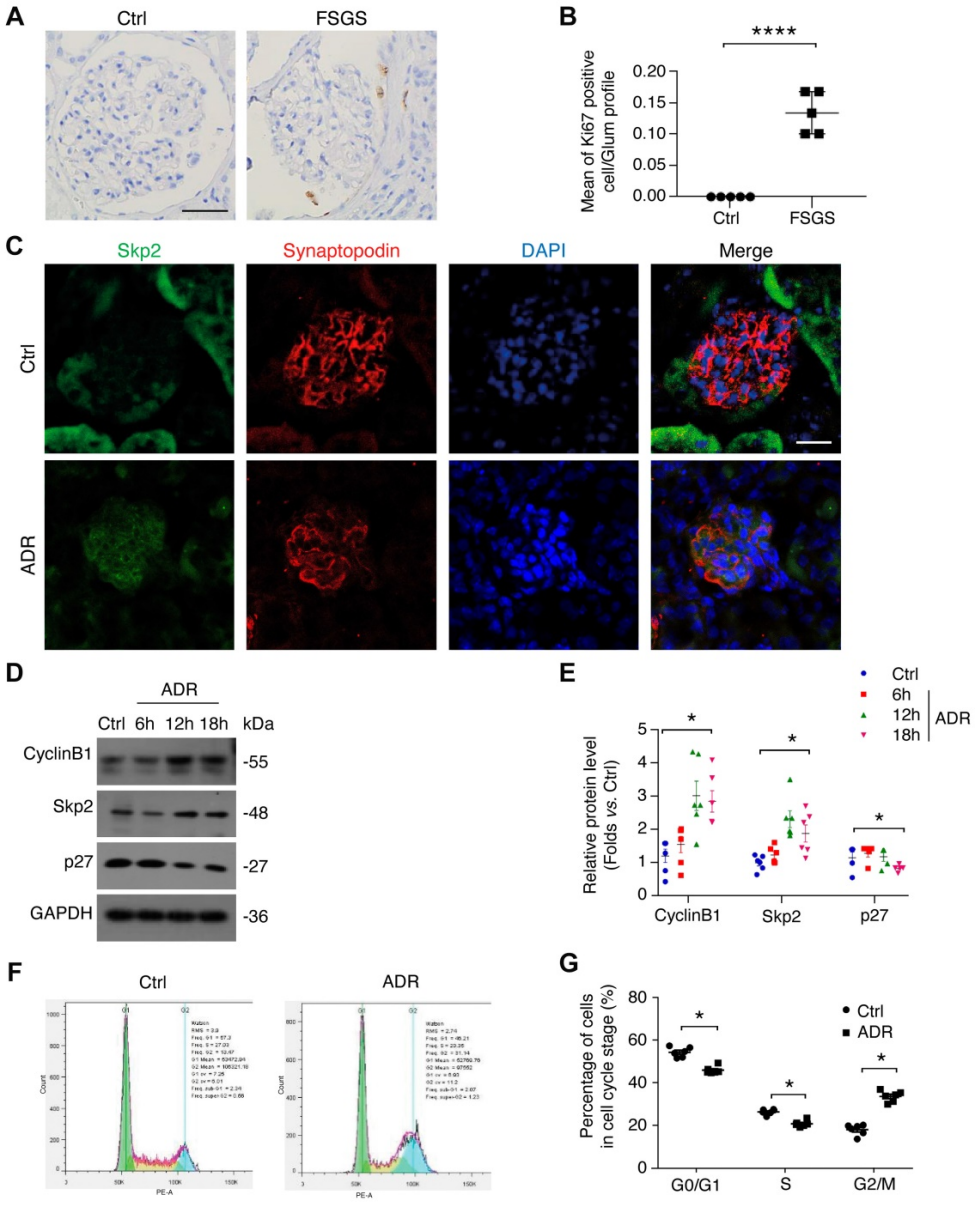
** ADR-induced cell cycle reentry in FSGS patients and disease models. (A, B)** Representative images (Scale bar=25μm) and summarized data of IHC staining highlighting Ki67-positive cells in patients with FSGS; *n*=5. **(C)** Representative confocal microscopic images showing Skp2 levels in the glomeruli of ADR mice, with synaptopodin used as a podocyte marker (Scale bar=25μm).** (D, E)** Representative western blot images and summarized data showing the expressions of cyclin B1, Skp2, and p27 in podocytes treated with 0.4 μg/ml ADR for indicated times; *n* = 6. **(F, G)** The cell cycle was analyzed by flow cytometry and the summarized data indicated the percentage of podocytes in each cell cycle stage; *n* = 5. ****P<0.0001 vs*.* Ctrl; *P<0.05 vs*.* Ctrl. **(B and G)** Statistical analysis was performed using two-tailed Student's *t*-test. **(E)** Statistical analysis was performed using one-way ANOVA with Dunnett's correction. FSGS, focal segmental glomerulosclerosis; ADR, adriamycin.

MAD2B negatively regulates APC/C and consequently modulates the process of cell cycle, so we firstly assessed the cell proliferation ability of the podocytes by evaluating the intensity of Ki67, a cell marker of proliferation. In the glomerular tuft from patients with FSGS, we found that the number of Ki67-positive cells was increased (*P*<0.0001) in comparison with control population (Figure 2A-B), indicating that cell cycle reentry had occurred. Secondly, we examined the substrate of APC/C and the regulators of the cell cycle in FSGS lesions. Dual IF staining showed that the podocytic Skp2 was elevated in the ADR-induced FSGS model, especially within the segments with prominent lesions (Figure 2C). The protein level of cyclin B1 and Skp2 was increased (*P*<0.05), while p27, a well-known cell cycle inhibitor, was decreased (*P*<0.05) in ADR-treated podocytes (Figure 2D-E). Thirdly, we evaluated the cell cycle by flow cytometry and found that the percentage of podocytes at G0/G1 (*P*<0.05) was reduced (Figure 2, F and G). These results indicate that along with elevated MAD2B expression, cell cycle reentry definitely occurs in patients with FSGS, animal models as well as ADR-treated podocytes.

### MAD2B deficiency ameliorates ADR-induced podocyte injury and cell cycle reentry in vitro

**Figure 3:**
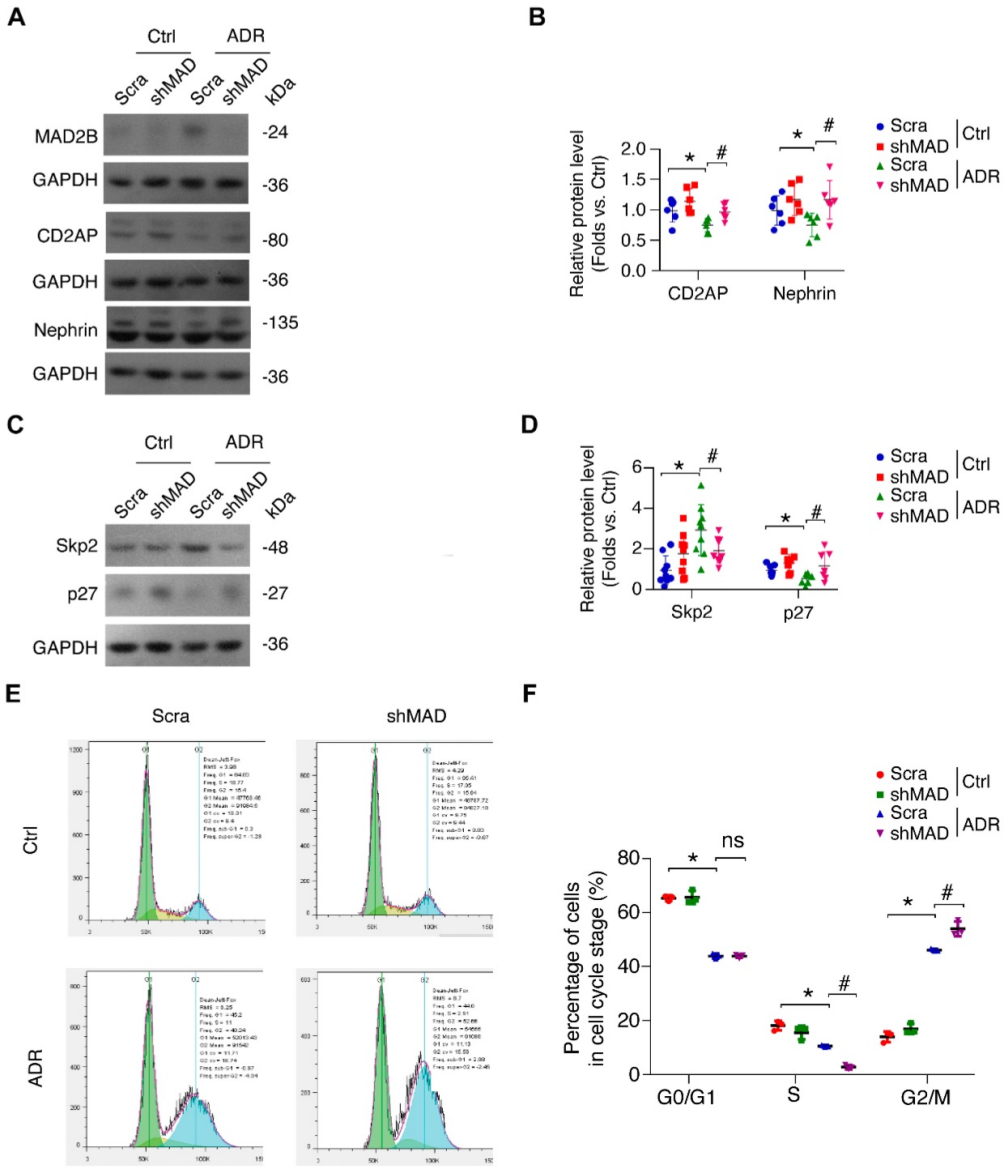
** MAD2B deficiency ameliorates ADR-induced podocyte injury and cell cycle reentry *in vitro*. (A, B)** Representative western blot images and summarized data showing that depletion of MAD2B by shRNA partially attenuates CD2AP and nephrin decline in ADR-stimulated podocytes; *n* = 6. **(C, D)** Representative western blot images and summarized data showing that MAD2B deficiency ameliorates ADR-induced cell cycle regulatory protein Skp2 and p27 alteration in podocytes; *n* = 6. **(E)** The percentage of podocytes in each cell cycle stage was measured by flow cytometry and** (F)** summarized data indicated that MAD2B deficiency partially mitigate ADR-induced cell cycle reentry; *n* = 5. Scra: scrambled shRNA; shMAD: MAD2B shRNA. *P<0.05 vs*.* Ctrl+Scra; ^#^*P*<0.05 vs*.* ADR+Scra. **(B, D and F)** Statistical analysis was performed using ANOVA with Dunnett's correction. ADR, adriamycin; MAD2B, mitotic arrest deficient-like 2B.

Nephrin is the major component of the slit diaphragm, mutations of which are known to result in congenital nephrotic syndrome of the Finnish type (CNF). Likewise, CD2AP is a cytoplasmic podocyte protein found to play a role in congenital nephrotic syndrome in mice and some human FSGS. Functionally, the loss of CD2AP and nephrin, pivotal podocyte molecules, was mitigated (*P*<0.05) by MAD2B shRNA transfection in ADR-treated podocytes (Figure 3A-B). Notably, MAD2B silencing attenuated the dysregulation of cell cycle regulatory proteins, including Skp2 (*P*<0.05) and p27 (*P*<0.05), with ADR stimulation (Figure 3C-D). Next, we analyzed the cell cycle status by flow cytometry and found that MAD2B knockdown increased the proportion of arrested cells in G2/M-phase (*P*<0.05) compared to scrambled shRNA group with ADR exposure (Figure 3E-F), avoiding disastrous division and following cell death. Taken together, MAD2B deficiency could ameliorate podocyte injury and cell cycle reentry initiated by ADR.

### Podocyte damage and proteinuria are relieved in podocyte-specific MAD2B knockout mice

**Figure 4:**
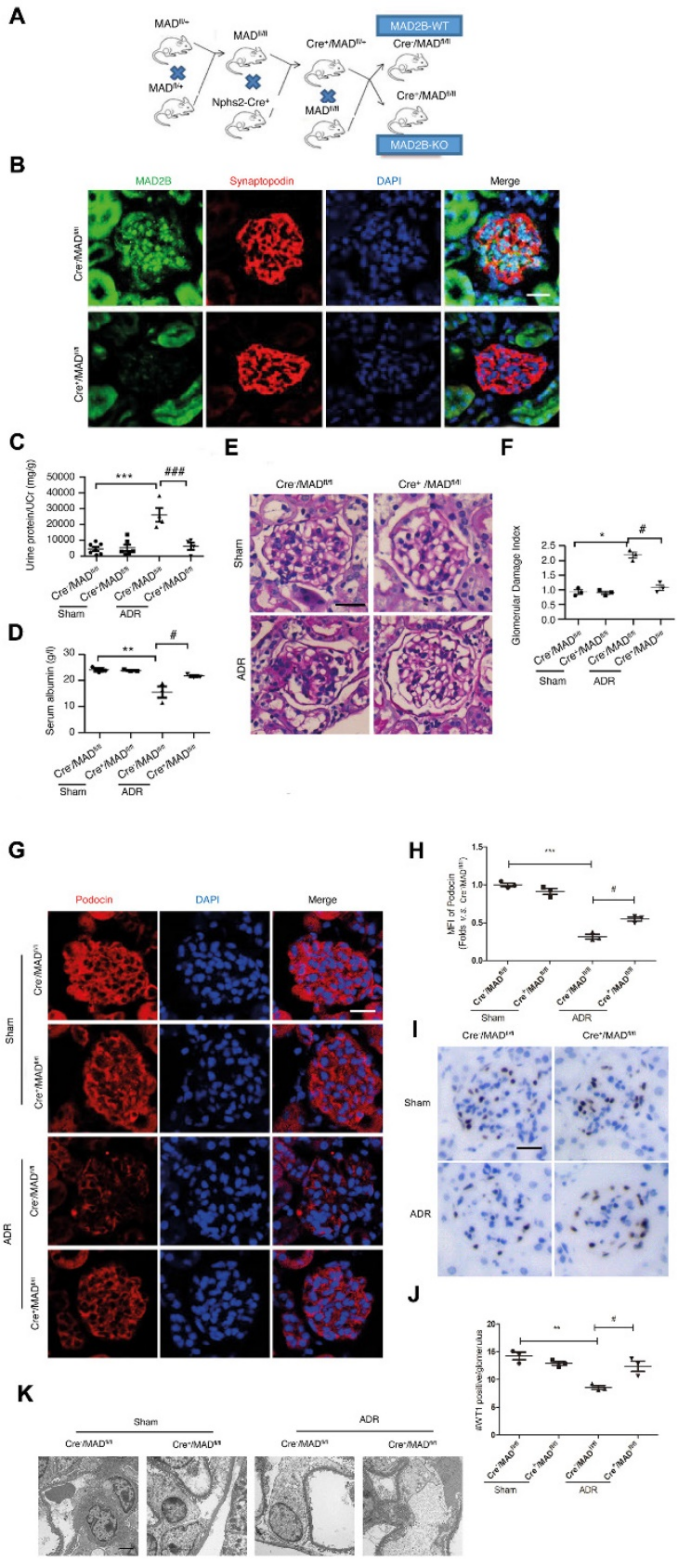
** Podocyte-specific deficiency of MAD2B ameliorates podocyte injury and proteinuria in ADR mice. (A)** Conditional knockout mice (Cre^+^/MAD2B*^fl/fl^*) were generated using the Cre-LoxP recombination system. **(B)** Representative confocal images of immunofluorescence staining of MAD2B and synaptopodin in the glomeruli of mice confirming that MAD2B was specially ablated in podocytes (Scale bar=25μm).** (C, D)** Summarized data showing the urine protein/creatinine ratio and serum albumin level of the experimental mice; *n* = 3-7. **(E)** Representative PAS staining images of glomeruli (Scale bar=25μm) and **(F)** summarized data showing the glomerular damage index (GDI) of four groups of mice; *n* = 3. **(G, H)** Representative images (Scale bar=25μm) and summarized data of immunofluorescence staining of slit diaphragm proteins indicating podocyte injury; *n* = 3. **(I, J)** Representative images (Scale bar=25μm) and summarized data of immunohistochemical staining of WT1 in the glomeruli of mice; *n* = 3.** (K)** Representative transmission electronic micrographs of Cre^-^/MAD*^fl/fl^* and Cre^+^/MAD*^fl/fl^* mice without or with ADR injection. (Scale bar=2μm). ADR-treated Cre^-^/MAD*^fl/fl^* mice revealed podocyte foot process effacement, which was significantly abrogated in Cre^+^/MAD*^fl/fl^* mice. Cre^+^/MAD*^fl/fl^*: podocyte-special knockout mice; Cre^-^/MAD*^fl/fl^* : WT littermates used as controls. *P<0.05 vs*.* Cre^-^/MAD*^fl/fl^*; **p< 0.01 vs*.* Cre^-^/MAD*^fl/fl^*; ***P<0.001 vs*.* Cre^-^/MAD*^fl/fl^*; ^#^*P*<0.05 vs*.* Cre^-^/MAD*^fl/fl^*+ADR; ^###^*P*<0.001 vs*.* Cre^-^/MAD*^fl/fl^*+ADR. **(C, D, F, H and J)** Statistical analysis was performed using one-way ANOVA with Dunnett's correction. PAS, Periodic acid-Schiff staining; MAD2B, mitotic arrest deficient-like 2B; ADR, adriamycin.

To directly evaluate the role of podocytic MAD2B in the ADR-induced FSGS model, conditional knockout mice were generated using the Cre-LoxP recombination system (Figure 4A). By IF staining, we confirmed that MAD2B was specifically deleted in podocytes (Figure 4B). Twelve-week-old podocyte-specific knockout mice (Cre^+^/MAD2B*^fl/fl^* mice) were intravenously administered a single dose of ADR (18 mg/kg) and observed for 6 wks before sacrifice. In Cre^+^/MAD2B*^fl/fl^* mice, ADR-triggered proteinuria (*P*<0.001) and hypoalbuminemia (*P*<0.05) (Figure 4C-D) as well as glomerulosclerosis (*P*<0.05) were much less prominent compared to Cre^-^/MAD2B*^fl/fl^* littermates (Figure 4E-F). In addition, podocin IF staining (Figure 4G-H) and WT1 immunostaining (Figure 4I-J) were performed to explore the podocyte injury and loss. The results showed that MAD2B deficiency could significantly ameliorate podocyte loss in ADR mice. Furthermore, using electron microscopy, we identified that the ADR-initiated loss and ultrastructural abnormalities of podocytes were significantly attenuated in Cre^+^/MAD2B*^fl/fl^* mice (Figure 4K).

### ATM kinase, a potential regulator of MAD2B, is markedly activated in FSGS mice and in ADR-treated podocytes

**Figure 5:**
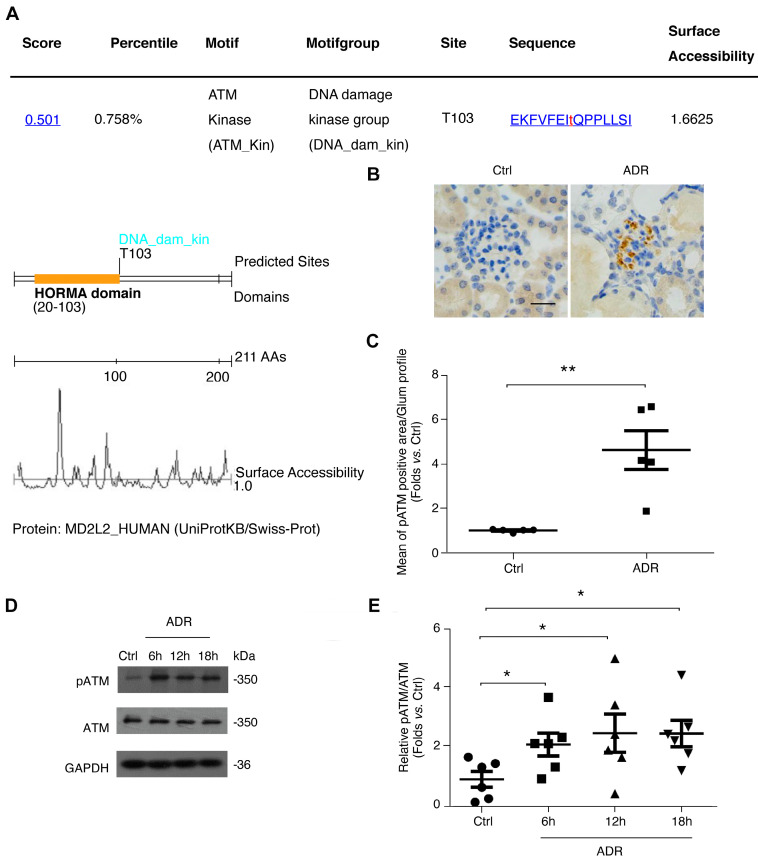
** ATM kinase, a potential regulator of MAD2B, is markedly activated in FSGS both *in vivo* and *in vitro*. (A)** Prediction of MAD2B phosphorylation site by Scansite indicated that MAD2B contains ATM S*/T*Q phosphorylation motif. **(B)** Representative photomicrograph of pATM density in the glomeruli of mice in each group (Scale bar=25μm) and **(C)** summarized data showing the mean of pATM positive area per glomerular profile; *n* = 6. **(D)** Representative western blot images and** (E)** summarized data showing ATM activation in ADR-treated podocytes; *n* = 6. Ctrl: control. *P<0.05 vs*.* Ctrl; **P<0.01 vs*.* Ctrl. **(C)** Statistical analysis was performed using two-tailed Student's *t*-test. **(E)** Statistical analysis was performed using ANOVA with Dunnett's correction. ADR, adriamycin; MAD2B, mitotic arrest deficient-like 2B; ATM kinase, ataxia-telangiectasia mutated kinase.

Considering the essential role of podocytic MAD2B in FSGS, we further explored the regulatory mechanisms responsible for its expression. First, we screened the putative interactor or regulator of MAD2B using Scansite software (http://scansite.mit.edu), and found that ATM kinase can phosphorylate MAD2B at Thr 103, highlighting the possibility of a modulatory role of ATM in MAD2B (Figure 5A). Moreover, the role of ATM in DNA repair through the 53BP1/MAD2B pathway supports the potential link between ATM kinase and MAD2B 10. It is well accepted that in terminally differentiated neuronal cells, ATM kinase is activated upon DNA damage, which usually leads to cell cycle reentry with subsequent cell death 18. Therefore, we evaluated the activity of ATM kinase, ATM S1981 phosphorylation (pATM), with ADR exposure. As shown in Figure 5B-C, ATM kinase was markedly activated not only in the glomeruli of ADR-treated mice (*P*<0.01) but also in podocytes with ADR exposure (*P*<0.05) (Figure 5D-E).

### ATM kinase ablation ameliorates cell cycle disorders and insults of podocytes in mice

**Figure 6:**
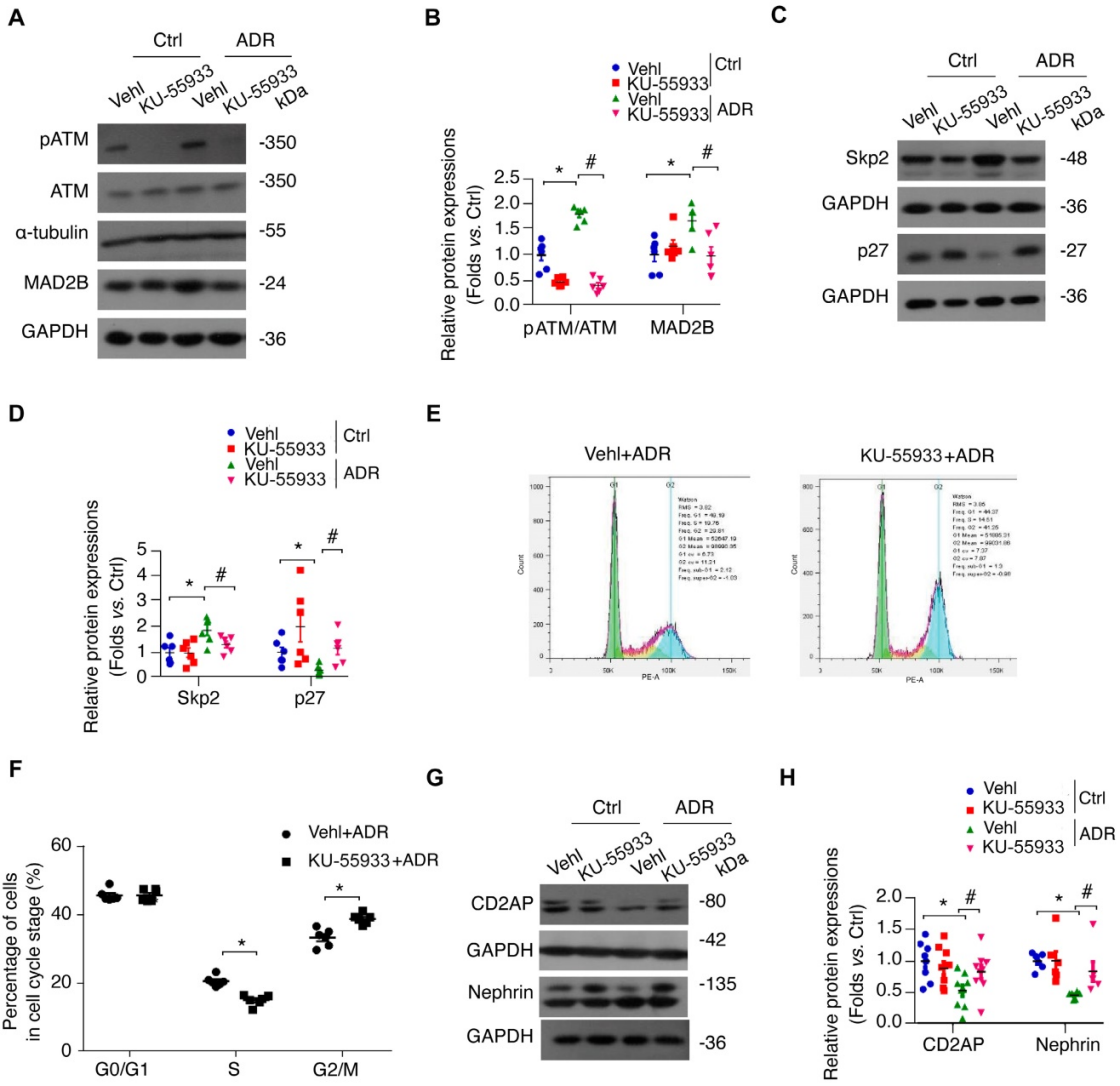
** Inhibition of ATM kinase can minimize MAD2B upregulation along with alleviated podocyte injury and cell cycle re-entry. (A)** Representative western blot images and **(B)** summarized data showing the ATM phosphorylation and MAD2B level in podocytes with ADR treatment. ATM kinase specific inhibitor, KU-55933 (10 μM), was pretreated for 2 h before ADR stimulation or not; *n* = 6. **(C)** Representative western blot images and **(D)** summarized data showing Skp2 and p27 levels; *n* = 6. **(E)** Representative images and **(F)** summarized data showing the percentage of cells in each cell cycle stage; *n* = 5. Vehl: Vehicle; *P<0.05 vs*.* Vehl+ADR. **(G)** Representative western blot images and **(H)** summarized data showing CD2AP and nephrin expression; *n* = 6. *P<0.05 vs*.* Vehl+Ctrl; ^#^*P*<0.05 vs*.* Vehl+ADR. **(B, D, and F)** Statistical analysis was performed using one-way ANOVA with Dunnett's correction. Statistical analysis was performed using two-tailed Student's *t*-test. ATM kinase, ataxia-telangiectasia mutated kinase; ADR, adriamycin.

**Figure 7:**
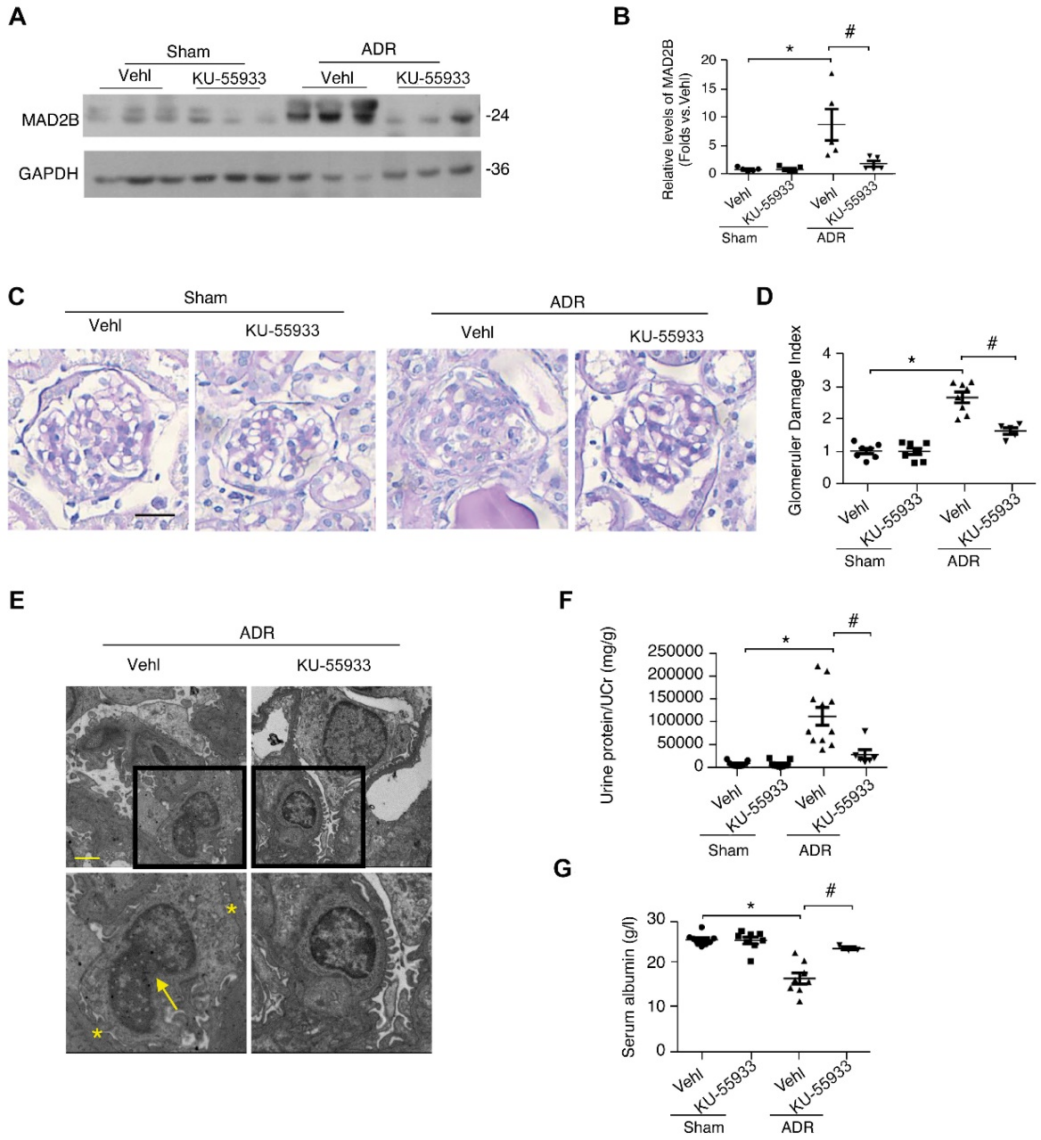
** ATM inhibition markedly alleviates proteinuria and podocyte injury in ADR-induced nephropathy. (A)** Representative western blot images and **(B)** summarized data showing the MAD2B protein level in kidney cortex of mice from different groups; *n* = 5. **(C)** Representative PAS staining images of glomeruli (Scale bar=25μm) and** (D)** summarized data showing the glomerular damage index (GDI) of the four groups of mice; *n* = 5-11. **(E)** Representative transmission electronic micrographs in different groups. The asterisks indicate typical podocyte foot process effacement. The arrow indicates bi-nucleated podocytes (Scale bar=2μm). **(F, G)** Summarized data showing the levels of proteinuria and serum albumin, respectively; *n* = 5-11. Vehl: vehicle; *p<0.05 vs*.* Vehl+Sham; ^#^*p*<0.05 vs*.* Vehl+ADR.** (B, D, F, and G)** Statistical analysis was performed using one-way ANOVA with Dunnett's correction. ATM kinase, ataxia-telangiectasia mutated kinase; MAD2B, mitotic arrest deficient-like 2B; PAS, Periodic acid-Schiff staining.

To further confirm the role of ATM in ADR-related podocyte cell cycle reentry, we used a specific ATM kinase inhibitor, KU-55933, to suppress the phosphorylation and activity of ATM. Strikingly, KU-55933 alleviated MAD2B elevation (*P*<0.05) caused by ADR stimulation (Figure 6A-B). Simultaneously, the alteration of Skp2 and p27, the well-known substrates of MAD2B and pivotal regulators of cell cycle, induced by ADR was partially reversed (*P*<0.05) by KU-55933 (Figure 6C-D). Moreover, flow cytometric analysis revealed that the ATM inhibitor increased podocytes arrested in G2/M-phase (*P*<0.05) (Figure 6E-F), avoiding disastrous division and following cell death. In addition, KU-55933 successfully prevented ADR-triggered podocyte dysfunction, as indicated by the recovery of nephrin (*P*<0.05) and CD2AP (*P*<0.05) expression (Figure 6G-H). Consistently, KU-55933 effectively suppressed the overexpression of MAD2B (*P*<0.05) provoking by ADR injection in mice (Figure 7A-B). The morphological abnormalities of FSGS were minimized by pretreatment with KU-55933 (Figure 7C-E), along with lower proteinuria (*P*<0.05) and elevated serum albumin (*P*<0.05) (Figure 7F-G). Therefore, our observation suggests that blocking ATM activation could effectively prevent or mitigate podocyte injury.

### ATM kinase phosphorylates MAD2B and regulates its ubiquitination in vitro

**Figure 8:**
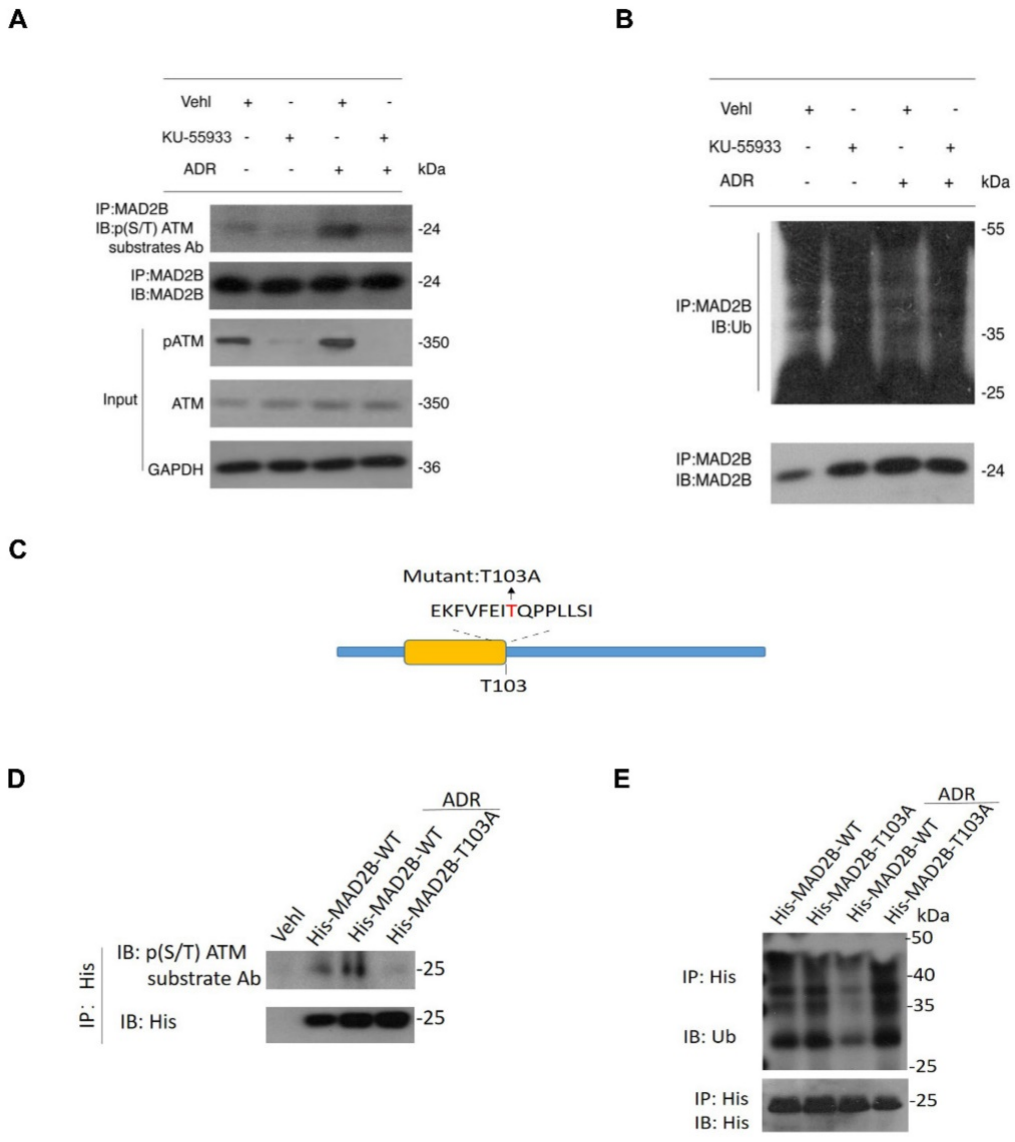
** The mechanism attributing to ATM-mediated MAD2B upregulation in vitro study. (A)** Immunoprecipitation of lysates from podocytes exposed to ADR (0.4 μg/ml) for 18 h with KU-55933 pretreatment or not, using an anti-MAD2B antibody followed by immunoblotting with a phospho-(S/T) ATM substrate antibody. **(B)** Cell lysates of podocytes, which were exposed to ADR with KU-55933 pretreatment or not, were incubated with MAD2B, and ubiquitination was detected by immunoblotting with anti-ubiquitin antibody.** (C)** Schematic showing the putative ATM phosphorylation site (T103) and mutagenesis. **(D and E)** Podocytes expressing His-MAD2B-WT or His-MAD2B-T103A constructs were treated with ADR (0.4 μg/ml) for 18 h, and immunoprecipitated using anti-His magnetic beads followed by immunoblotting with a phospho-(S/T) ATM substrate antibody and anti-ubiquitin antibody, respectively. Ub-MAD2B represents ubiquitinated MAD2B. ATM kinase, ataxia-telangiectasia mutated kinase; MAD2B, mitotic arrest deficient-like 2B; ADR, adriamycin.

Earlier, we identified that Thr103 as a potential ATM phosphorylation site in MAD2B, moreover, it is described that almost all the substrates of ATM kinase contain an indispensable motif termed S*/T*Q 19. Here, immunoprecipitation data showed that MAD2B was recognized by a pan-phospho-(S/T) ATM substrate antibody, which further supports that ATM is a kinase involved in MAD2B phosphorylation. Importantly, the intensity of MAD2B phosphorylation was significantly upregulated with ADR treatment, and blocking ATM kinase using KU-55933 successfully reversed the abnormal MAD2B phosphorylation (Figure 8A). Because phosphorylation has a close link with ubiquitination and is a prerequisite in protein degradation, we analyzed the post-translational ubiquitination level of MAD2B in ADR-treated podocytes. Our findings indicate that ADR-triggered MAD2B deubiquitination could be effectively dampened by KU-55933 pretreatment (Figure 8B). To elucidate the mechanism of MAD2B phosphorylation and degradation, additional studies using site-directed mutagenesis were performed. Mutation of the Thr 103 ATM phosphorylation site (Figure 8C) in MAD2B abrogated the phosphorylation (Figure 8D) and reduced ubiquitylation of MAD2B (Figure 8E).

## Discussion

Over the past few decades, incidences of end-stage renal disease due to FSGS are increasing 20. It is well recognized that cell cycle disorder followed by podocyte loss is a cardinal cellular event for the pathogenesis of FSGS, which is different from minimal change disease (MCD). In FSGS, podocyte injury is almost entirely irreversible, whereas this lesion is repairable in MCD. Podocytes are highly differentiated “post-mitotic” cells with fine assembled interdigitating foot processes 21. Under certain stimuli, the surviving podocytes tend to reenter the cell cycle and synthesize DNA to increase their size, thus becoming hypertrophic, which may compensate for GBM denudation and podocyte loss. However, hypertrophic podocytes do not have the ability to assemble the correct mitotic spindle and complete mitosis 8, 22, 23. Once they are divided, the outcome is fatal. Morphologically, bi/multi-nucleated podocytes are not uncommon in podocytopathies, especially in FSGS. In addition, emerging evidence shows that invalid mitosis consequently leads to cell death, accelerating podocyte loss and glomerulosclerosis. Mechanistically, the role of cell-cycle proteins, such as cyclin 23, 24, is quite different in podocytes and other terminal differentiated cells comparing to its role in proliferative cells. Thus, the molecular mechanisms accounting for cell cycle control in podocytes need to be further elucidated.

MAD2B can depress APC/C to modulate the cell cycle 11. Previous studies from our and other groups illustrated that MAD2B is attributed to either physiological or pathophysiological events in the kidneys 14, 25, 26. In the present study, we verified that MAD2B is upregulated in the glomeruli of patients with FSGS and a murine model with aberrant expression of cell cycle regulators. Consistently, flow cytometry analysis demonstrated that ADR initiates cell cycle reentry in podocytes. Thus, our findings suggest that a relatively low level of MAD2B is required to maintain a “post-mitotic” homeostasis of podocytes, and elevated MAD2B expression may contribute to cell cycle reentry of podocytes. Indeed, knockdown of MAD2B could reverse ADR-induced cell cycle reentry of podocytes as well as FSGS lesions and proteinuria in a murine FSGS model. Therefore, our data confirmed that the overexpression of MAD2B results in cell cycle reentry and podocyte injury in FSGS.

Next, with the aid of bioinformatics, we identified ATM kinase as the upstream modulator of MAD2B, and ATM phosphorylated MAD2B at Thr103 site. Accumulating evidences supports that ATM kinase is a sensor protein of double-stranded DNA breaks and is involved in cell cycle checkpoint control 27. Physiologically, ATM is held inactive as a dimer or a higher-order multimer. Once upon cellular stress, it is autophosphorylated at Ser1981, causing dimer dissociation and ATM activation, which endows its kinase competence. To date, there is a lack of data on the role of ATM in the kidneys. However, in post-mitotic neurons, cell cycle reentry and its related cell death can be successfully interrupted by blocking ATM 18. In the current study, we found that under ADR exposure, ATM is activated by Ser1981 phosphorylation. In addition, interrupting ATM kinase could depress MAD2B level and alleviate the cell cycle disturbances of podocytes. Considering that during ADR insult podocytes re-entered the cell cycle, cells might die from mitotic catastrophe along with the cell cycle progression. In the study of flow cytometry, only viable podocytes were harvested and tested. Thus cell cycle analysis data are biased by the effect of podocyte loss, presenting as underestimated proportion of podocytes in G0/G1 phase and G2/M phase arrest with less aberrant mitosis during MAD2B knockdown or ATM inhibition. Concurrently, ATM blockage significantly ameliorated proteinuria and podocyte injury in a murine FSGS model. Taken together, ATM is a putative kinase regulating MAD2B expression and is implicated in ADR-induced cell cycle reentry of podocytes.

Phosphorylation and ubiquitination are two quite important post-translational modifications and they have a remarkably close association. Phosphorylation usually affects protein ubiquitination and stability 28. For example, activated ATM kinase phosphorylates the tumor suppressor FBXO31 at Ser278 and Gln279, inhibiting its ubiquitination and resulting in FBXO31 accumulation 29. Our results showed that besides mediating MAD2B phosphorylation, ATM synchronously mitigated its ubiquitination during ADR treatment, which was successfully reversed by ATM inhibitor. Therefore, these findings collectively imply that ATM activation could phosphorylate MAD2B and hamper its ubiquitination, which results in MAD2B accumulation and ensuing APC/C depression and cell cycle disorder.

In summary, the APC/C inhibitor MAD2B is normally maintained at low level in mature podocytes. During FSGS, ATM phosphorylates MAD2B and depresses its ubiquitination, which consequently leads to MAD2B overexpression and cell cycle disturbance. Targeting ATM-MAD2B could protect podocytes against cell cycle reentry and the ensuing lesion. These data elucidate the mechanisms of FSGS via cell cycle abnormalities, and provide a promising therapeutic target for FSGS.

## Abbreviations

FSGS: focal segmental glomerulosclerosis
MAD2B: mitotic arrest deficient 2-like protein 2
APC/C: anaphase-promoting complex/cyclosome
ATM: ataxia-telangiectasia mutated
GBM: glomerular basement membrane
Cdh1: cadherin1
CDC20: cell division cycle 20
Skp2: S-phase kinase-associated protein 2
ADR: adriamycin
PAS: periodic acid-Schiff
DMSO: dimethyl sulphoxide
GDI: glomerular damage index
CD2AP: CD2 associated protein
IHC: immunohistochemical
IF: immunofluorescence
pATM: ATM S1981 phosphorylation
CNF: congenital nephrotic syndrome of the Finnish type
WT1: Wilms tumor gene
MCD: minimal change disease
